# Biophysical and Computational Studies of the vCCI:vMIP-II Complex

**DOI:** 10.3390/ijms18081778

**Published:** 2017-08-16

**Authors:** Anna F. Nguyen, Nai-Wei Kuo, Laura J. Showalter, Ricardo Ramos, Cynthia M. Dupureur, Michael E. Colvin, Patricia J. LiWang

**Affiliations:** 1 Departments of Molecular Cell Biology and Chemistry and Chemical Biology, and the Health Sciences Research Institute, University of California Merced 5200 North Lake Rd, Merced, CA 953402, USA; aankirskaia@ucmerced.edu (A.F.N.); nicolekuo2010@gmail.com (N.-W.K.); lshowalter@ucmerced.edu (L.J.S.); rramos4@ucmerced.edu (R.R.); mcolvin@ucmerced.edu (M.E.C.); 2 Department of Chemistry & Biochemistry, University of Missouri-St. Louis, St. Louis, MO 63121, USA; dupureurc@umsl.edu

**Keywords:** chemokine binding protein, chemokine analog, anti-inflammation, 35K, vCCI, vMIP-II, MIP-1β/CCL4, molecular dynamics

## Abstract

Certain viruses have the ability to subvert the mammalian immune response, including interference in the chemokine system. Poxviruses produce the chemokine binding protein vCCI (viral CC chemokine inhibitor; also called 35K), which tightly binds to CC chemokines. To facilitate the study of vCCI, we first provide a protocol to produce folded vCCI from *Escherichia coli* (*E. coli*.) It is shown here that vCCI binds with unusually high affinity to viral Macrophage Inflammatory Protein-II (vMIP-II), a chemokine analog produced by the virus, human herpesvirus 8 (HHV-8). Fluorescence anisotropy was used to investigate the vCCI:vMIP-II complex and shows that vCCI binds to vMIP-II with a higher affinity than most other chemokines, having a *K*_d_ of 0.06 ± 0.006 nM. Nuclear magnetic resonance (NMR) chemical shift perturbation experiments indicate that key amino acids used for binding in the complex are similar to those found in previous work. Molecular dynamics were then used to compare the vCCI:vMIP-II complex with the known vCCI:Macrophage Inflammatory Protein-1β/CC-Chemokine Ligand 4 (MIP-1β/CCL4) complex. The simulations show key interactions, such as those between E143 and D75 in vCCI/35K and R18 in vMIP-II. Further, in a comparison of 1 μs molecular dynamics (MD) trajectories, vMIP-II shows more overall surface binding to vCCI than does the chemokine MIP-1β. vMIP-II maintains unique contacts at its N-terminus to vCCI that are not made by MIP-1β, and vMIP-II also makes more contacts with the vCCI flexible acidic loop (located between the second and third beta strands) than does MIP-1β. These studies provide evidence for the basis of the tight vCCI:vMIP-II interaction while elucidating the vCCI:MIP-1β interaction, and allow insight into the structure of proteins that are capable of broadly subverting the mammalian immune system.

## 1. Introduction

Protein–protein interactions are critical for many aspects of biological and immunological function. Of particular interest are virally-encoded proteins that undermine the immune system, often by having the ability to promiscuously bind many targets, and therefore, help the virus evade immune surveillance. One such system targeted by viruses is the chemokine system, in which virally encoded proteins disrupt the chemokine receptor/ligand interaction [1]. Chemokines (chemotactic cytokines) are a class of small secreted proteins that mediate immune cell chemotaxis as part of the inflammatory response. There are about 18 human chemokine receptors that are activated upon binding to their cognate chemokine ligand [2]. About 50 chemokine ligands are known, spanning 4 sub-families. The two major subfamilies are CC chemokines (named for the adjacent Cys near the N-terminus of the protein) and CXC chemokines (named for having an intervening amino acid between the conserved N-terminal Cys). In general, CC chemokines (named numerically as CCL1; chemokine ligand 1, etc.) bind to and activate cognate receptors on the surfaces of monocytes, macrophages and T-cells, and these receptors are numerically named as CC receptors (e.g., CCR1, CCR2). CXC chemokines tend to have cognate receptors on the surface of neutrophils [2], with receptors such as CXCR1. Chemokines can sometimes bind multiple receptors, and receptors often have more than one cognate ligand, although CC chemokines are restricted to CC chemokine receptors, and CXC chemokines have their own CXC receptors. 

Because of the central nature of the chemokine system in activating and localizing immune cells, subversion of the process may be useful to a virus. Several types of chemokine-binding proteins (CKBPs) have been identified (reviewed in [3]), including those that bind chemokines from multiple subfamilies, such as Myxoma-T7 (M-T7) from myxoma [4], M3 from γ-herpesvirus-68 [5,6,7,8], and the poxvirus-encoded smallpox virus-encoded chemokine receptor (SECRET) domain [9]. These proteins have gained interest as inflammation inhibitors, due to their ability to bind to pro-inflammatory proteins.

One of the most potent inhibitors of chemokine action is the poxvirus-encoded protein vCCI (viral CC chemokine inhibitor; also called 35K). This approximately 240 amino acid protein binds 80 CC-chemokines across several species, about 20 of which have nanomolar affinity to this inhibitor [10,11]. The protein sequence across several poxviruses shows high identity, and the structures from cowpox [12], rabbitpox [13], and mousepox [14] reveal a beta sandwich with a binding face containing several key negatively charged amino acids, as well as a long acidic loop between beta strands 2 and 3. We have carried out structural studies of vCCI in complex with MIP-1β (also called CCL4 [13]), which revealed details of the interaction between vCCI and the chemokine, including several close contacts that are critical for binding. Mutagenesis of vCCI/35K by others, in vitro and in vivo, has confirmed the importance of several of the residues suggested by the structure, including E143 and the acidic loop [14,15]. Mutagenesis studies on the chemokines themselves have also been carried out by us and others and indicate that several evolutionarily conserved, positively charged residues are important for binding to vCCI/35K [11,16,17]. In our work, with a variety of eotaxin mutants (CCL11 [11]), we showed that eotaxin’s binding to vCCI was dependent on the presence of several basic residues in the chemokine. 

Viruses have also evolved the ability to interfere with the chemokine system by producing chemokine homologs, small proteins that mimic the chemokine’s ability to bind a chemokine receptor, thus blocking the native chemokine. Herpesvirus HHV-8 encodes several such chemokine analogs; of particular interest is the protein vMIP-II (virally encoded macrophage inflammatory protein-II), which has about 40% identity with the human CC chemokine MIP-1β, and has been shown to bind and antagonize several CC chemokine receptors (CCR1, CCR2, CCR5, though it can agonize CCR3 and CCR8), as well as at least one CXC receptor (CXCR4) [18,19,20]. This range of receptor binding is much greater than a typical chemokine. We have previously studied vMIP-II to elucidate its ability to bind glycosaminoglycans, and have shown that, in solution, it is a soluble monomer with a fold similar to that of MIP-1β [21,22]. Due to its nanomolar affinity to, and broad ability to bind to chemokine receptors [18,22], vMIP-II has engendered interest as an anti-inflammatory agent, with some success in rat studies involving ischemic stroke [23], spinal cord injury [24], and kidney transplant rejection [25].

vCCI and vMIP-II are therefore complementary proteins, the former having evolved to bind a large variety of CC chemokines, and the latter having evolved to be a prototypical chemokine ligand with the ability to bind many receptors. While we have studied these proteins in complex with their natural ligands [11,13,22,26], we developed the hypothesis that a significant amount of insight could be obtained by determining whether a tight complex could be formed by these proteins. In other words, we set out to study the complex between an “ideal” chemokine binding protein (vCCI) and an “ideal” chemokine homolog (vMIP-II). Due to the broad action of these proteins, an understanding of these powerful viral tools may be helpful in designing strategies to manipulate or control immune responses, and could be applicable to fields ranging from autoimmunity to traumatic brain injury.

We present a technique for producing rabbitpox vCCI from *Escherichia coli* (*E. coli*.), as well as experimental and molecular dynamics analysis of the vCCI:vMIP-II complex and the vCCI:MIP-1β complex, comparing these two complexes to explore the differences in binding between the virus-encoded chemokine analog and a natural human chemokine [13]. Our results show that the affinity of vCCI to vMIP-II is higher than that between vCCI and natural chemokines [11,18] and suggest explanations for this high affinity, as well as for previously-reported functional results. 

## 2. Results

### 2.1. Folded Viral CC Chemokine Inhibitor(vCCI)/35K Can Be Produced from E. coli

Despite interest in the mechanism of affinity of vCCI, and for its possible use as a therapeutic, it has been relatively time consuming to produce in vitro, since bacterial expression results in unfolded protein. High expression of proteins from this family have been described from yeast [11,12,13], baculovirus [14,17], and an antibody fragment crystallizable region(Fc)-linked vCCI was produced from 293T mammalian cells [15]. Each technique is useful, but the lack of an *E. coli* expression protocol has limited study of the protein, and in particular, limited the ability of investigators to easily make a wide range of mutants. We have expressed vCCI from *E. coli*, and show that the protein can successfully be refolded. Briefly, the cells are disrupted in 6 M guanidine hydrochloride at pH 8.0 under reducing conditions, and purified with a nickel chelating column. After further reducing agent is added, the solution is slowly added to 20× volume of a cold refold buffer containing l-arginine, sucrose, and glutathione, and incubated for 1 day. The solution is then dialyzed at pH 7.4, followed by addition of a protease to allow cleavage of the fusion tag. Final purification is carried out on an anion-exchange column (see Materials and Methods for details).

**Figure 1:**
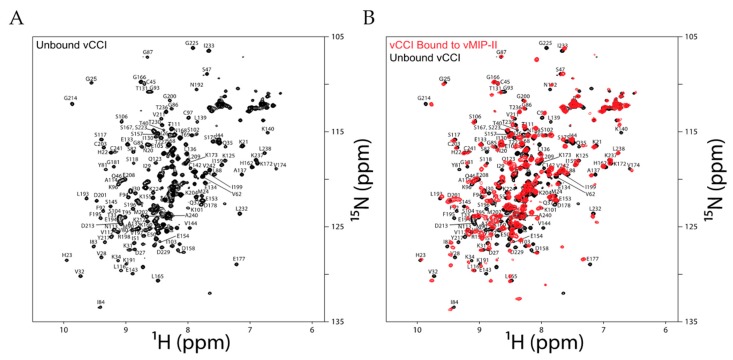
(**A**) ^1^H-^15^N heteronuclear single quantum coherence (HSQC) spectrum of unbound ^15^N-labeled vCCI in 100 mM NaCl, 20 mM NaOP (sodium phosphate) pH 7.0, measured at 37 °C; (**B**) Overlay of the ^1^H-^15^N HSQC spectra of free ^15^N-labeled viral CC chemokine inhibitor (vCCI) (black) and ^15^N vCCI:^14^N vMIP-II (red) with a ratio of 1:3, measured under the same conditions as in (**A**). The concentration of vCCI was 50–60 μM.

The final product of refolding and purification results in a ^15^N heteronuclear single quantum coherence (HSQC) spectrum of vCCI/35K that is essentially identical to that produced from *Pichia pastoris* yeast expression (Figure 1A and Figure S1). Further, this protein forms a complex upon addition of chemokine analog vMIP-II (Figure 1B), showing its functionality.

### 2.2. vCCI:vMIP-II Produce a High Affinity Complex

vCCI has a remarkable ability to bind almost all chemokines from the CC subfamily, and a qualitative measure of its binding with herpesvirus encoded vMIP-II has been reported [10]. To more quantitatively investigate the affinity between vCCI and vMIP-II, we used isothermal titration calorimetry, in which vMIP-II was titrated into a solution of vCCI. This technique can provide several thermodynamic parameters, and often a dissociation constant. Analysis of the titration data indicated that the *K*_d_ of the complex was below 1 × 10^−10^ M (Table S1). This is the lowest detectable limit of the instrument, and so an alternate method was used to obtain a more accurate binding constant. 

**Figure 2:**
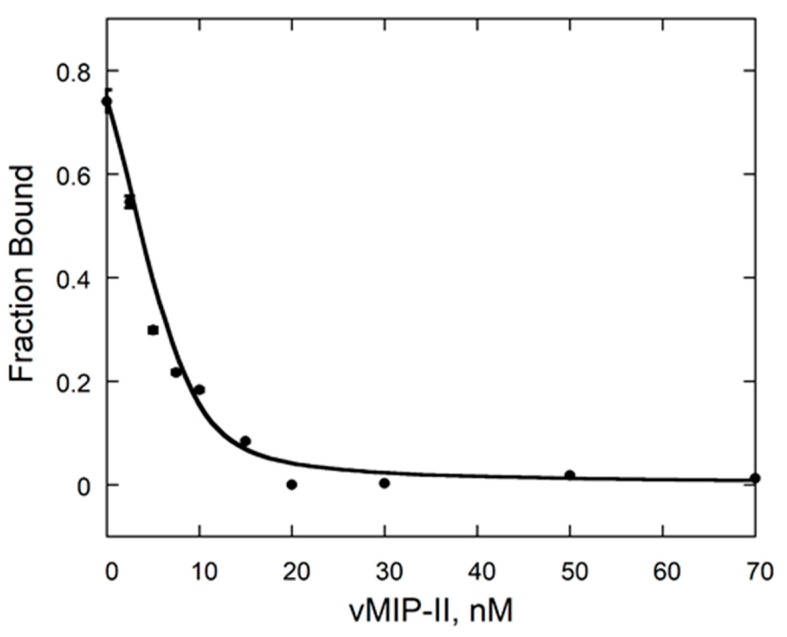
Competition fluorescence anisotropy of the vCCI:vMIP-II interaction. vMIP-II was added to a complex of vCCI with fluorescently labeled eotaxin-1 (CCL11) as in [11]. Error bars are shown, but are sometimes within the size of the data point.

An alternative method of obtaining affinity involves a competition technique in which vCCI is bound to a fluorescently labeled chemokine (eotaxin-1/CCL11), and the competitor (vMIP-II in this case) is titrated into the solution, with the resulting change in fluorescence anisotropy providing the dissociation constant for the interaction [11]. This showed a *K*_d_ of 0.06 ± 0.006 nM for the vCCI:vMIP-II interaction (Figure 2). This is among the tightest measured vCCI:chemokine interactions.

### 2.3. Changes in Chemical Shift Suggest vCCI:vMIP-II Interaction Is Similar to Other vCCI:Chemokine Complexes

**Figure 3:**
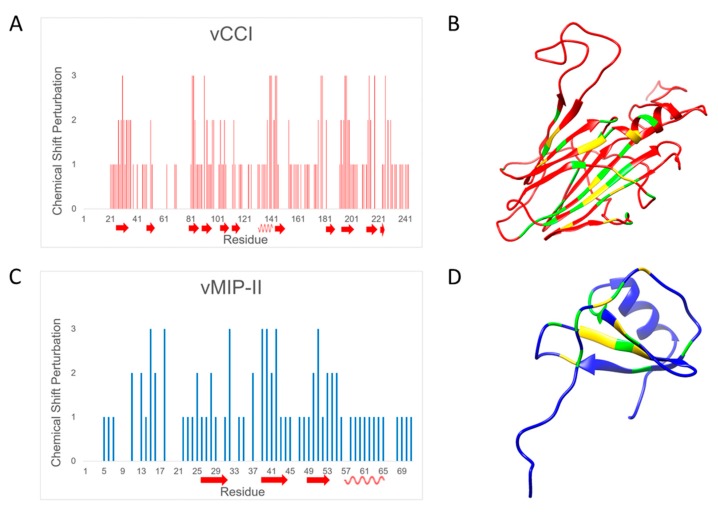
Changes in chemical shift upon complex formation. (**A**) Changes in chemical shift in vCCI upon binding to vMIP-II. Arrows represent beta strands while spiral lines represent alpha helices. See Table 1 for definitions of “0”, “1”, “2”, “3”; (**B**) Structure showing changes in vCCI (Protein Data Bank code 2FFK upon binding vMIP-II. Green indicates greater than average, up to 1 standard deviation away from the average; yellow indicates over 1 standard deviation away from the average; red indicates below one standard deviation or the peak had not been discernable; (**C**) Changes in chemical shift in vMIP-II upon binding to vCCI. Secondary structure is shown by arrows and spiral lines, as in (A). See Table 1 for definitions of “0”, “1”, “2”, “3”; (**D**) Structure showing those changes in vMIP-II upon binding vCCI (Protein Data Bank code 1VMP). Green indicates greater than average, up to 1 standard deviation away from the average; yellow indicates over 1 standard deviation away from the average; blue indicates below one standard deviation or the peak had not been discernable. All structure figures were prepared by using UCSF Chimera (UCSF Resource for Biocomputing, Visualization, and Informatics, San Francisco, CA, USA) [28].

To determine the amino acids that are likely involved in the vCCI:vMIP-II complex, a comparison of chemical shift changes between the free and bound forms of both vCCI/35K and vMIP-II was carried out. In the case of vCCI, ^15^N HSQC spectra in the free and bound form using ^15^N labeled vCCI (with non-isotopically labeled vMIP-II) were measured and compared, to determine the level of peak movement upon complex formation. (Assignments of the unbound vCCI were obtained from [27] and Biological Magnetic Resonance Bank (BMRB) databank 6809.) Chemical shift changes in the ^15^N vMIP-II HSQC spectrum upon binding non-isotopically labeled vCCI/35K were also determined. In cases where unambiguous assignment of the peak in the bound spectrum was not possible, conservative assessments of peak movements were made, indicating that actual peak movement could be greater than shown. Figure 3 shows residue-by-residue chemical shift change. See Table 1 for definitions of chemical shift perturbation categories.

As shown in Figure 3A, the areas of greatest chemical shift change for vCCI/35K upon binding to vMIP-II are located in the region of amino acids in the 80’s, 140’s, and 190’s, with changes also observed in the 30’s, 170’s–180’s and 220’s. These areas are shown on the structure of vCCI/35K (shown without vMIP-II) in Figure 3B, and indicate a binding surface similar to those shown previously [11,13,14,27], comprised of negative charges (D141, E143, as well as likely the negatively charged loop in the 52–77 region that is not clearly assignable, likely due to flexibility) as well as interaction in the early 80’s region. Figure 3C indicates that vMIP-II interacts with vCCI/35K using residues from its N-loop region (residues 12–19), and with the second beta strand in the 30’s region, as well as with residues in the early 50’s. Figure 3D shows vMIP-II with presumed interacting regions highlighted.

### 2.4. Molecular Dynamics Simulations on vCCI:vMIP-II

**Figure 4:**
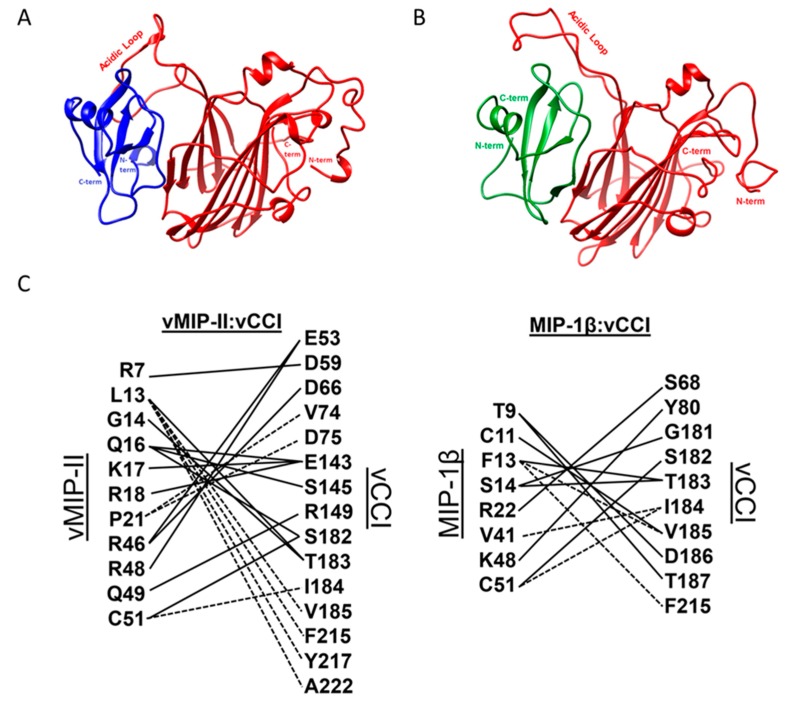
Complexes after 1 μs molecular dynamics simulation of vCCI:chemokine. For all figures, vCCI is in red ribbon, and the bound chemokine is either blue ribbon (vMIP-II) or green ribbon (MIP-1β). (**A**) Structure of vCCI:vMIP-II after 1 μs trajectory; (**B**) Structure of vCCI:MIP-1β in complex after 1 μs trajectory; (**C**) Interactions between residues of vCCI and vMIP-II, as well as vCCI and MIP-1β. Hydrogen bonds (solid lines) are shown if they appear in at least 50% of the last 300 ns of the molecular dynamics simulation. Dashed lines indicate non-hydrogen-bond interactions between residues. These are defined as residues whose access to solvent is occluded upon complex formation at least 50% of the time, and that are within 2.8 Å of the partner residue on the other protein in at least 50% of the structures sampled every 20 ns for the final 500 ns of the trajectory.

To further clarify the structural components and the likely interacting surfaces of the complex, and to gain insight into the extraordinarily tight binding between vCCI/35K and vMIP-II, we carried out molecular dynamics (MD) simulations to create 1 microsecond (μs) trajectories of both the vCCI:MIP-1β complex and the vCCI:vMIP-II complex. Both trajectories are based on the reported vCCI:MIP-1β structure (Protein Data Bank (PDB) ID: 2FFK), but for MIP-1β, the residues were changed to reflect the wild type chemokine, as opposed to the triple mutant used in that structure determination [13]. The vCCI:vMIP-II trajectory was created from the same complex structure, but by computationally superimposing the vMIP-II structure onto the MIP-1β chain to minimize the average difference between the corresponding Cα backbone atoms ([13]; Figure 4A,B and Figure S2). (A third simulation was also included that used the 2FFK structure directly, keeping MIP-1β as a triple mutant (K45A/R46A/K48A) instead of changing it to wild type. However, this third simulation is not emphasized in this work; see the Materials and Methods section for more details on the simulations.)

Analysis of the secondary structure of the complexes during the trajectory shows that all of the α-helices and β-sheets are preserved though out the 1 microsecond runs for all three complexes (see Figure S3). The root-mean-square deviation (RMSD) in the position of the backbone Cα atoms for the entire complex is less than 1 nm over the entire runs and the RMSD for the individual vCCI and MIP-1β chains is less than 0.8 nm, indicating no gross protein disordering over the simulation, although there are highly flexible regions in both the vCCI and the chemokines. The residue-level fluctuations (RMSF) are the root-mean square fluctuations of each residue around the average protein structure for a trajectory. The RMSF values plotted vs sequence location and calculated for the final 750 ns of the trajectories are shown in Figure S4. These show significant fluctuations of the vCCI N-terminal residues in all three complexes, as well as in the vCCI loop at residues 52–77. As described below, the loop acts as an “arm” that folds down on the bound chemokine. The MIP-1β structures show significant fluctuations at both the N- and C-termini, while vMIP-II shows fewer fluctuations at its termini.

**Figure 5:**
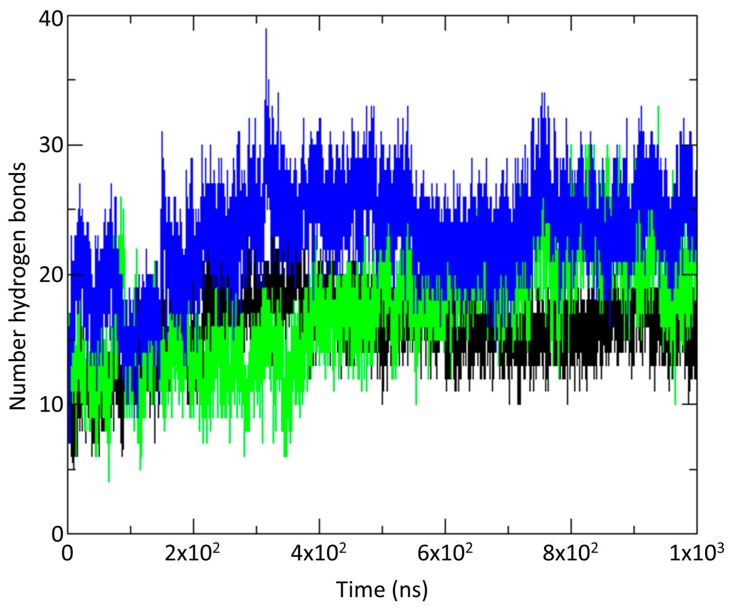
Number of interstrand (vCCI:chemokine) hydrogen bonds observed throughout the molecular dynamics (MD) trajectory for vCCI:vMIP-II (blue); vCCI:MIP-1β (green) and vCCI:MIP-1β-K45A/R46A/L48A (black).

Figure 5 plots the total number of interstrand (vCCI:chemokine) hydrogen bonds over the 1 microsecond simulation for each of the three complexes. The vCCI:vMIP-II complex has significantly more interstrand hydrogen bonds than either of the MIP-1β complexes while the wild type MIP-1β has more than the mutant. Figure 4C shows the hydrogen bonds formed in the vCCI complex with both vMIP-II and MIP-1β.

We analyzed the effect of individual residues in vCCI and the chemokine ligand on solvent exposure, using the server-based program PDBePISA [29,30,31]. The program computes the solvent accessible surface area for both the complex and the computationally separated fragments, and reports the solvent-exposed surface area of each residue in the separated proteins, and the amount of area that is buried when the complex is formed. This analysis was performed every 10 nanoseconds for the final 500 nanoseconds of the simulation, for a total of 51 structures analyzed per complex. The three complexes show different total amounts of buried surface area, as shown in Table 2. Additionally, specific residue–residue contacts vary between the three different complexes. Figure S5 shows interactions of residues that are occluded during the simulation upon complex formation at least 50% of the time, and that are within 2.8 Å of the partner residue in at least 50% of the structures sampled every 20 ns for the final 500 ns of the trajectory.

A comparison of the dynamics trajectories of vCCI/35K binding to vMIP-II and to MIP-1β shows some striking differences, in particular, revealing several possible interactions that may account for the approximately 10-fold tighter binding for vMIP-II to vCCI/35K. First, at the end of the simulation, the total buried surface area for vMIP-II in complex is 1528 Å^2^, while the buried surface area for MIP-1β in complex is 1392 Å^2^. Second, as shown in Figure 5 and Figure 4C, during the time course of the trajectory, vCCI/35K shows an overall larger number of hydrogen bonds with vMIP-II than with MIP-1β. Third, the flexible, negatively charged loop in the 52–77 region of vCCI (between beta strands 2 and 3) makes more contact, including a larger number of hydrogen bonds over the course of the trajectory, with vMIP-II than with MIP-1β. And finally, during the 1 μs trajectory, vMIP-II shows overall more interactions with vCCI than does MIP-1β, in particular, at the N-terminus of the chemokine where a large portion of that region of vMIP-II lays across the vCCI binding face, while the MIP-1β N-terminus does not.

**Figure 6:**
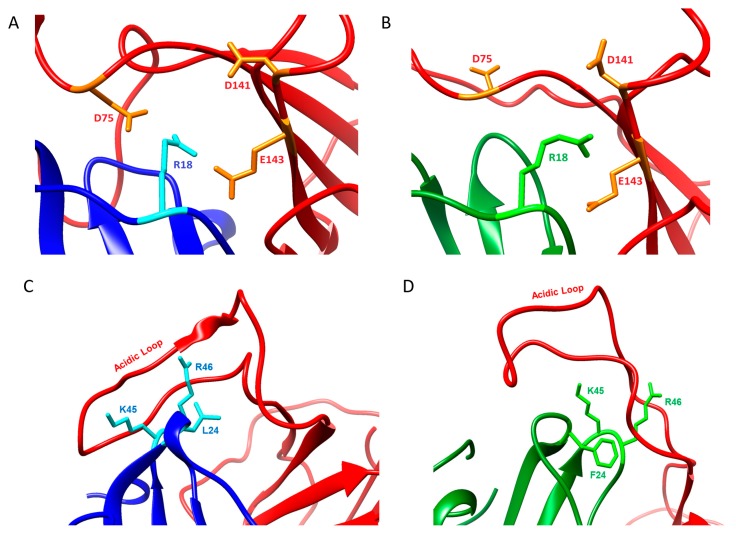
Interactions between vCCI and vMIP-II or MIP-1β. vCCI is in red ribbon and the bound chemokine is either blue ribbon (vMIP-II) or green ribbon (MIP-1β). (**A**) A close-up of the interaction between R18 of vMIP-II (cyan) and D75, D141, E143 of vCCI (orange); (**B**) A close-up of the interaction between R18 of MIP-1β (bright green) and D75, D141, E143 of vCCI (orange); (**C**) A close-up of the interaction between the β2 and β3 loop of vCCI (red) and K45 and R46 of vMIP-II (cyan). L24 of vMIP-II is also indicated in cyan; (**D**) A close-up of the interaction between the β2 and β3 loop of vCCI (red) and K45 and R46 of MIP-1β (bright green). F24 of MIP-1β is also indicated in bright green.

The vCCI:vMIP-II trajectory shows several individual interactions that illuminate aspects of their binding and complementary interactions, including significant contact throughout the trajectory between residues E143 on vCCI/35K and residue R18 in vMIP-II (Figure 6A); and interaction between the negatively charged loop between strands β2 and β3 in vCCI/35K with K45/R46 in vMIP-II (Figure 6C). A similar trajectory is seen for vCCI:MIP-1β, with MIP-1β residue R18 showing interactions with E143, as well as D141 of vCCI (Figure 6B). However, vMIP-II’s R18 residue also shows interaction with vCCI residue D75 for almost half the time steps in the trajectory, while no such interaction is observed with MIP-1β. The trajectories also show both vMIP-II and MIP-1β have their 24/45/46 position residues clustered together, and interacting with the vCCI loop between β2 and β3, but the interaction is much more extensive in the vCCI:vMIP-II complex (Figure 6C,D and Figure 4C). The N-terminus of each of the chemokines also behaves very differently in the trajectory. vMIP-II shows considerably more interaction with vCCI throughout its N-terminus for much of the trajectory, while the MIP-1β trajectory shows an N-terminus that does not appear to interact consistently with vCCI, with main contacts to the binding partner not starting until residue 8. In total, the simulation results point to possible reasons why vCCI shows different binding constants to various partners.

## 3. Discussion

The ability to modulate the immune system, and in particular, to reduce the inflammatory response, has great potential in health and medicine. Protein therapeutics have been approved for this purpose [32,33], and investigation has continued into other potential sources of anti-inflammatory proteins. Both herpesviruses and poxviruses have evolved to produce proteins that subvert the mammalian chemokine system, and these include both chemokine binding proteins as well as chemokine homologs [3,34,35]. The current work investigates the unusually high affinity interaction between the vCCI/35K chemokine binding protein from rabbitpox, and vMIP-II, a chemokine analog from herpesvirus HHV-8, with a combination of biophysical and molecular dynamics techniques. 

vCCI was successfully produced and purified from *E. coli*. This fairly efficient procedure will greatly expand the range of experiments that can be carried out with vCCI, from quickly making large quantities of the protein (and any desired variants) for X-ray crystallography, to inexpensive isotopic labeling that can lead to a variety of NMR experiments, including a full structure determination. Isothermal titration calorimetry indicated a high affinity for the vCCI:vMIP-II complex, and this was confirmed by fluorescence anisotropy, which revealed a *K*_d_ of 0.06 nM ± 0.006 nM that is significantly lower than the *K*_d_ observed for vCCI with other chemokines using the same method [11]. Other groups have investigated the binding constant of vCCI with various chemokines using other methods, including early qualitative work that suggested that vCCI/35K bound vMIP-II more tightly than most other chemokines [10]. Others have used a scintillation proximity assay [35] and surface plasmon resonance [14,16,17] to show that vCCI/35K binds a variety of chemokines at levels ranging from sub-nanomolar to 20 nM.

Analysis of chemical shift perturbation by NMR indicates that vCCI/35K interacts with vMIP-II using residues similar to those that have been shown to be important in binding by vCCI to other chemokines, MIP-1β/CCL4, and eotaxin-1/CCL11 [11,13], such as acidic residues in the 141/143 area. Similarly, chemical shift perturbation of vMIP-II upon binding vCCI/35K shows chemical shift changes in generally known areas, including the region near R18, as well as the area near the so-called N-loop of the chemokine, where hydrophobic L13 is located (Figure 3). While NMR chemical shift perturbation is a powerful tool, there are two main drawbacks. First, while a perturbation suggests a locus for protein–protein interaction, and one can infer regions of interaction between proteins, it does not confirm a pairwise interaction with the binding partner. Second, 2D ^15^N HSQC spectra can be ambiguous in terms of assigning peaks upon movement. To resolve ambiguity would require ^13^C-labeling of the protein(s) and a series of 3D NMR experiments [11]. Therefore, we chose to pursue molecular dynamics simulations, which provide a high resolution “movie” (within certain approximations) of the structure and motions of all atoms in the protein and surrounding solvent, and can delineate specific interactions and provide insight into differences in affinity.

Atomistic classical molecular dynamics (MD) is a well-established tool for studying protein structure and dynamics [36]. In typical protein MD, the motions of all atoms are simulated using empirical force fields that approximate the forces due to bonded and non-bonded interactions. The resulting output is a high-resolution series of atomic motions that can be analyzed to characterize the structure and dynamics of the protein, and infer the energy causes of the observed behavior. With modern computers and MD software, it is feasible to routinely run simulations of moderately large proteins (including a solvation shell of water and ions) for microsecond timescales, with the largest published MD simulations reaching millisecond times [37]. The accuracy is limited by the approximate nature of the force field and the limitation that bonds are not broken or formed during the simulations (including protonation and deprotonation of acid and base sites), but MD has been shown able to accurately predict protein properties, such as the folded conformation of small proteins [38].

In the investigation of the vCCI:vMIP-II complex, a 1 μs MD trajectory was run, providing great insight into likely interactions that were not observable and/or confirmable by our NMR experiments to this point. In general, the hypothesis that these two viral proteins may be a near-ideal binding pair is supported by the MD trajectories, which show that that vCCI:vMIP-II complex has a larger buried surface area (including the vMIP-II’s N-terminus) and a greater number of hydrogen bonds throughout the trajectory, including more interactions between the chemokine and the negatively charged flexible loop of vCCI than the vCCI:MIP-1β complex. The MD simulations also provide context for specific regions of interaction that may be useful in general for a vCCI:chemokine complex. For example, D141 and E143 in vCCI were observed to contact R18 in the vCCI:MIP-1β structure [13], and this R18 was found to be critical for vCCI/35K binding in other chemokines [11,13,16,17]. However, mutational studies on vCCI/35K showed E143 to be more important than D141 [15]. The MD trajectory provides an explanation, showing significant, continuous interaction between E143 (vCCI/35K) and R18 (vMIP-II), while almost no close interaction across the trajectory is observed with D141. In the vCCI:MIP-1β trajectory, significant hydrogen bonding interaction (although below the 50% threshold for Figure 4C) is observed between R18 of the chemokine and both E143 and D141 of vCCI, although the interaction with E143 predominates.

**Figure 7:**
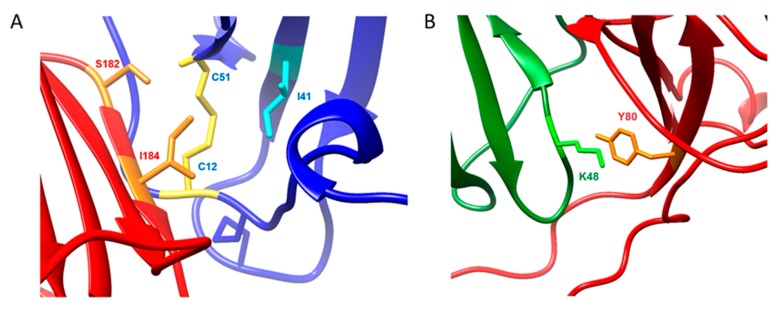
Interactions illuminated by molecular dynamics simulation of the vCCI:MIP-1β and vCCI:vMIP-II complex. vCCI is in red ribbon in both instances, bound vMIP-II is in blue ribbon, and bound MIP-1β is in green ribbon. (A) Interaction between I184 of vCCI (orange) and I41 of vMIP-II (cyan), as well as S182 of vCCI (orange) with the backbone of C51 of vMIP-II (yellow). C12 of vMIP-II is also indicated in yellow; (B) Interaction between Y80 of vCCI (orange) and the backbone of K48 of MIP-1β (green).

The MD trajectory also provides a possible explanation for other unexplained mutational results. In the original structure of the complex between vCCI/35K and MIP-1β /CCL4, it was observed that both Y80 and R89 in vCCI/35K appeared close in space to the 48^th^ position of MIP-1β [13]. In many chemokines, this position contains a large, basic residue that could be expected to both sterically and electrostatically clash with those groups. It had been noted that mutation of this residue to the smaller Ala48 increased affinity for a similar chemokine MCP-1/CCL2 [16,17]. Indeed, MIP-1β was mutated from a Lys to an Ala at that position in the structure, and that mutation was attributed to tighter binding to vCCI. In an attempt to design a vCCI/35K that was better able to bind chemokines, White et al. mutated each Y80 and R89 to Ala in vaccinia vCCI/35K, hypothesizing that a smaller, uncharged residue in these positions would better interact with the large basic residue of a chemokine [15]. Interestingly, while the vCCI/35K R89A mutation did lead to a better chemokine binding ability, Y80A completely abolished the activity of the protein. The MD trajectory of Y80 in the vCCI:MIP-1β complex shows the tyrosine side chain of vCCI consistently forming a hydrogen bond with the backbone nitrogen of Lys48 of MIP-1β (Figure 4C and Figure 7B). This Y80 hydrogen bond was not consistently observed in the vCCI:vMIP-II trajectory, although the trajectory shows consistent contact between these residues (Figure S5). In either case, the Y80 in this crowded area of the protein shows little motion and appears to be holding open the negatively charged loop in vCCI. The Y80 residue in vCCI has also been mutated by Arnold et al., who replaced tyrosine with arginine. This mutation did also lead to loss of chemokine binding ability, although in this case, the cause is likely placing a basic Arg on vCCI near the Arg48 of a chemokine [14].

The MD trajectory has helped reveal several interesting interactions that NMR alone had difficulty explaining. Arnold et al. observed that the mutation of residues S182 and I184 (using the present vCCI numbering) resulted in substantial loss of binding activity, especially for I184 [14]. However, previous chemical shift assignments for vCCI with other chemokines [11,13] as well as the current work with vMIP-II, are unable to quantify the shifts to these residues in vCCI. The MD trajectory, however, reveals an interaction between VCCI I184 and vMIP-II Ile41 and Cys51, seemingly to help anchor vCCI to the chemokine throughout the trajectory (Figure 7A); this interaction is also seen in the vCCI:MIP-1β trajectory, explaining large chemical shift changes in chemokines in the region 39–42. vCCI Ser182, meanwhile, appears to have formed a hydrogen bond to the backbone N-H of C51 in both vMIP-II and MIP-1β. This may explain the large chemical shift changes to that region of the spectra. This was also observed in the chemical shift changes of eotaxin in the vCCI:eotaxin complex [11]. 

Overall, a combination of biochemical, biophysical, and computational experiments have been used to provide a comprehensive explanation of the basis for the high affinity interaction between vCCI/35K and vMIP-II. These proteins each exemplify a highly evolved mechanism to mimic and/or subvert the mammalian immune system, and an understanding of their interaction will be useful in both their development as possible therapeutics and in general protein design for immunomodulation.

## 4. Materials and Methods

### 4.1. Protein Purification

#### 4.1.1. Purifying vCCI from *E. coli*

The gene sequence encoding the rabbit pox vCCI was slightly modified by PCR to allow for proper cleavage by enterokinase, which does not cut efficiently near proline. DNA coding for Met-Pro in the first two amino acids was replaced with DNA coding for Ala-Met-Ala. The resulting gene was cloned into the pET32a vector utilizing the restriction sites *NcoI* and *HindIII*. The plasmid was transformed into *E. coli* BL21 (DE3) (Novagen, Madison, WI, USA) competent cells and expressed in Luria broth or minimal media with ^15^NH_4_Cl as the sole nitrogen source. Protein production was induced when the absorbance at 600 nm reached 0.70–0.75 with the addition of isopropyl β-d-1-thiogalactopyranoside (IPTG) to 1mM and incubated with shaking at 22 °C for 20 h. The cells were then harvested by centrifugation at 4200× *g*, 4 °C for 12 min and supernatant was discarded.

The cell pellet was resuspended in 6 M guanidine hydrochloride, 200 mM NaCl, and 50 mM Tris (pH 8.0) and was lysed by three passages through a French press and then centrifuged at 27,000× *g* for 1 h. The supernatant was decanted and 15 mM β-mercaptoethanol (β-ME) was added and allowed to stir at room temperature for 2 hours to reduce. The solution was then loaded onto a nickel chelating column (Qiagen, Hilden, Germany) equilibrated with the resuspension buffer containing 15 mM βME after a thorough 0.3 M imidazole wash to remove unbound nickel. The column containing the bound vCCI was washed with 10 column volumes of resuspension buffer containing 15 mM βME, and then with 10 column volumes of wash buffer (6 M Guanidinium chloride, 200 mM NaCl, 15 mM βME, 80 mM NaOP, pH 7.2). Proteins were eluted from the column using 6 M guanidine hydrochloride, 200 mM NaCl, and 60 mM NaOAc, pH 4. Fractions containing the eluted protein were identified by absorbance at 280 nm and then pooled together. βME was then added to a concentration of 25 mM. The fractions were allowed to stir for one hour at room temperature, followed by stirring for 12 hours at 4 °C overnight. 

The protein was then refolded by dropwise addition to 20x volume of ice-cold refolding buffer (9.6 mM NaCl, 0.4 mM KCl, 2 mM CaCl_2_, 2 mM MgCl_2_, 550 mM l-arginine hydrochloride, 400 mM sucrose, 3 mM reduced glutathione (GSH), 0.3 mM oxidized glutathione (GSSG), 50 mM Tris, pH 8), and then allowed to stir 24 h at 4 °C. The solution was dialyzed 4 times into 4 liters 200 mM NaCl, 2 mM CaCl_2_, 20 mM Tris, pH 7.4 buffer at 4 °C.

To cleave the thioredoxin fusion tag from the purified protein, the samples were incubated for 12 hours at 4 °C with 650 nM of the protease enterokinase. The samples were then dialyzed 4 times into 4 L of 20 mM Bis-Tris, 50 mM NaCl pH 7.1 and then passed through a 0.2 μm nylon filter to then be purified on a HiTrap™ Q HP Column (GE Healthcare Life Sciences, Chicago, IL, USA) using a gradient from 50 mM NaCl, 20 mM Bis-Tris pH 7.1 to 1 M NaCl, 20 mM Bis-Tris pH 7.1, to separate the cleaved tag from vCCI. The fractions were analyzed on an SDS-PAGE gel to confirm purity and then fractions containing vCCI were concentrated using the Amicon concentrators (Millipore, Billerica, MA, USA), and buffer was changed to 100 mM NaCl, 20 mM NaOP pH 7.0 with 0.02% NaN_3_ for NMR studies. 

#### 4.1.2. Expression and Purification of vMIP-II

The gene for vMIP-II was placed into a pET28 vector and transformed into *Escherichia coli* BL21 (DE3) (Novagen, Madison, WI, USA) competent cells and expressed in either minimal media with ^15^NH_4_Cl as the sole nitrogen source for ^15^N-labeled samples or Luria Broth for ^14^N-labeled samples. Protein production was induced by adding IPTG to 1 mM and incubated with shaking at 37 °C for 5 h.

The cell pellet was resuspended in 6 M guanidine hydrochloride, 200 mM NaCl, 10 mM benzamidine, 50 mM Tris (pH 8.0) and were lysed by French press and then centrifuged at 27,000× *g* for 1 h. The soluble portion was then loaded onto a nickel chelating column (Qiagen, Hilden, Germany) equilibrated with the resuspension buffer. Proteins were eluted from the column using a pH gradient with 6 M guanidine hydrochloride, 200 mM NaCl, 60 mM NaOAc (pH 4) followed by addition of 10 mM βME while stirring for 2 hours at room temperature. The proteins were then refolded by dropwise addition into 10× volume of refolding buffer (550 mM l-arginine hydrochloride, 200 mM NaCl, 1 mM EDTA (Ethylenediaminetetraacetic acid), 1 mM reduced glutathione (GSH), 0.1 mM oxidized glutathione (GSSG), 50 mM Tris, pH 8), and then allowed to stir overnight at 4 °C. The solution was dialyzed three times into 4 L of 200 mM NaCl, 20 mM Tris pH 8 buffer at 4 °C.

To cleave the Small Ubiquitin-like Modifier (SUMO) fusion tag from the purified protein, the samples were incubated for 12 hours with 100 nM of the Ubl-specific protease 1 (ULP-1). The protein solution was then centrifuged to remove precipitated material and added onto a second nickel chelating column (Qiagen, Hilden, Germany), with flow-through containing the cleaved vMIP-II being collected. The samples were then dialyzed and purified on a C_4_ reversed-phase chromatography column (Vydac, Hesperia, CA, USA), using an acetonitrile gradient. The fractions were analyzed on an sodium dodecyl sulfate polyacrylamide gel electrophoresis (SDS-PAGE) gel to confirm purity and lyophilized in a Labconco freeze-dry system.

The proteases used in these purifications were produced and purified in our laboratory as briefly described: ULP1 or enterokinase protease were proteins were expressed in LB medium using a pET-28b vector and the cells were collected and French pressed. The ULP1 protease from the supernatant was purified using a nickel chelating column [39]. The enterokinase was found in the inclusion body and resuspended in 6M guanidinium buffer before being purified using a nickel chelating column. Enterokinase was then dialyzed in buffer to allow for refolding and tested for activity through self-cleavage of the fusion tag (manuscript in preparation). 

Proteins used in fluorescence anisotropy studies were purified as specified in [11]. vCCI for these experiments were made using a gene encoding rabbitpox virus vCCI cloned into pPIC9K plasmid and then transformed into *Pichia pastoris* strain SMD1168 (Invitrogen, Carlsbad, CA, USA) and purified as previously described [13].

### 4.2. Nuclear Magnetic Resonance (NMR) Spectroscopy

All NMR samples were made in 20 mM sodium phosphate buffer, 100 mM NaCl with 10% D_2_O, 5 μM 2,2-dimethyl-2-silapentane-5-sulfonic acid (DSS), and 0.02% NaN_3_, with a final pH of 7.0. ^15^N labeled lyophilized vMIP-II was resuspended into 5 mM NaOP buffer, pH 2.8 in order to dissolve the protein, and then 10 μL was added to 340 μL NMR buffer (either alone or with 150 uM ^14^N-vCCI) for final vMIP-II concentration of 50-60 μM. ^14^N-vMIP-II was also resuspended into 5 mM NaOP, pH 2.8, and 10 μL was added to 340 μL NMR buffer containing ^15^N-labeled vCCI, for a final concentration of 150 μM VMIP-II in the sample. vCCI was exchanged into NMR buffer as explained in the above sections, with final concentrations for ^15^N samples being 50–60 μM. 

All HSQC NMR data were acquired on a four-channel 600 MHz Bruker Avance III spectrometer (Bruker Corp, Billerica, MA) equipped with a (GRASP II) gradient accessory and a (TCI) cryoprobe with an actively shielded Z-gradient coil. Spectra with ^15^N-labeled vMIP-II were measured at 25 °C; spectra with ^15^N-labeled vCCI were measured at 37 °C. The chemical shift was referenced relative to internal DSS [40]. The data were processed using NMRPipe [41] and analyzed using PIPP [42] (Available online: https://spin.niddk.nih.gov/bax/software/NMRPipe/). For HSQC spectra, sweep width = 8474.576(^1^H) and 1766.784 Hz (^15^N), with 1280 points in ^1^H and 128* (256 total) points in ^15^N. 

The weighted average chemical shift change of the ^1^H and ^15^N resonances for each residue upon binding was calculated using the equation [43]:

(1) $$\Delta \delta_{obs}\;=\sqrt{\frac{{{\Delta \delta }_{H}}^{2}+{\left(\frac{\Delta \delta_{N}}{5}\right)}^{2}}{2}}$$

where Δδ_H_ and Δδ_N_ are the chemical shift changes of the ^1^H and ^15^N dimensions, respectively. Here, the Δδ_obs_ is the difference between bound and free form of ^15^N-labeled complexes. Due to lack of ^13^C labeling, some bound peak identifications were estimates; to be conservative, the nearest residue without a clear origin were assumed to belong to the residues in question.

### 4.3. Isothermal Titration Calorimetry (ITC) 

A Nano ITC Low Volume isothermal titration calorimeter (TA Instruments, New Castle, DE, USA) was loaded with degassed 10 uM vCCI in 20 mM NaOP, 100 mM NaCl, pH 7.0 and water in the reference cell. Twenty 2.5 μL injections of 100 μM vMIP-II also in 20 mM NaOP, 100 mM NaCl, pH 7.0 were then injected at 300 second intervals, with a 350 rpm stirring speed. Baseline selection, buffer-into-buffer blank was subtracted from the data, and peak-by-peak manual integration was performed using NanoAnalyze software (TA Instruments, New Castle, DE, USA). The data for an independent binding site model was provided by the software. The *K*_d_ was below detectable limits (10^−10^ nM). 

### 4.4. Fluorescence Anisotropy

Fluorescence anisotropy experiments were carried out in three independent experiments as described in [11], at 25 °C and pH of 7.0 utilizing a Photon Counting (PC1) spectrofluorimeter and VINCI software (ISS, Champaign, IL, USA), with an excitation wavelength of 497 nm and emission wavelength of 524 nm. The obtained data were then fit to a system of mass conservation equations as well as the following equation:

(2) $$\theta =\frac{{\left[L\right]}_{free}\;\times \;K_{a}}{1\;+\;{\left[L\right]}_{free}\;\times \;K_{a}}$$

where θ is the fraction of bound eotaxin-K63C, [L]_free_ is the concentration of unbound vCCI, and *K*_a_ is the association constant for the complex. 

For the competitive binding experiment, a 1:1 ratio of the vCCI:eotaxin-K63C (both proteins were prepared and purified as described in [11], with eotaxin-K63C labeled with fluorescein-5-maleimide) complex was prepared at a concentration of 8 nM. 500 μL of this complex was then mixed with varying amounts of unlabeled vMIP-II and incubated 30 minutes at 25 °C. Anisotropy measurements were taken and the values were normalized so that 1 represents the 100% bound state. The resulting data were fit using Scientist software (Micromath, Salt Lake City, UT, USA) to a system of equations described previously [44,45].

### 4.5. Molecular Dynamics

All-atom molecular dynamics was performed on three vCCI:MIP complexes. All three complex structures were based on the NMR structure of the VCCI:MIP-1β complex (PDB: 2FFK), which has three mutations from the wild type MIP-1β sequence. We recreated the original wild type structure by in silico editing of the 2FFK experimental structure. The vCCI:vMIP-II starting structure was created from the vCCI:MIP-1β structure by computationally superimposing the experimental vMIP structure (PDB code 1VMP) on the MIP-1β chain, to minimize the average difference between the corresponding Cα backbone atoms (see Figure S2). The net charge (−11 vCCI:vMIP-II, −26 vCCI:MIP-1β mutant, −23 vCCI:MIP-1β wild type) on the complexes was neutralized by adding Na+ ions and additional Na^+^/Cl^−^ pairs (~60) were added to yield an ion concentration of approximately 70 millimolar. After short equilibration runs, a full 1 microsecond of MD simulation was run using Gromacs 5.0.7 [46,47,48] using the NPT ensemble, the Verlet cutoff scheme and a 2 fs timestep. All bonds to hydrogen were constrained to their equilibrium length using the LINCS algorithm [49]. Temperature was maintained at 300K using the Bussi et al. thermostat [50] and pressure at 1 bar using the Parrinello-Rahman barostat [51]. The simulations were performed using the AMBER99SB-ILDN force field for the protein [52] and the TIP3P water model [53]. 

### 4.6. Figure Preparation

All structure figures were prepared by using UCSF Chimera (UCSF Resource for Biocomputing, Visualization, and Informatics, San Francisco, CA, USA) [28].

## Figures and Tables

**Table 1:** Definitions of the chemical shift perturbation categories in Figure 3.

Chemical Shift Perturbation	Definition	vCCI Chemical Shift	vMIP-II Chemical Shift
0	No confirmable change	No peaks visible	No peaks visible
1	Less than or equal to average	Δδ_obs_ ≤ 0.045	Δδ_obs_ ≤ 0.100
2	Greater than average, up to one standard deviation above average	0.045 < Δδ_obs_ ≤ 0.086	0.100 < Δδ_obs_ ≤ 0.178
3	Greater than one standard deviation above average	Δδ_obs_ ≥ 0.086	Δδ_obs_ ≥ 0.178

Δδobs: the difference in chemical shift between bound and free form of ^15^N-labeled complexes, as defined in Methods.

**Table 2:** Buried surface area between vCCI and chemokine variants averaged over 51 structures analyzed during the final 500 ns of simulation.

Complex	vCCI Buried Surface Area (Å^2^)	Chemokine Buried Surface Area (Å^2^)
vCCI:vMIP-II	1473	1528
vCCI:MIP-1β wild type	1355	1392
vCCI:MIP-1β K45A/R46A/K48A (variant used in 2FFK structure determination)	1020	1060

## Supplementary Materials

Supplementary materials can be found at www.mdpi.com/1422-0067/18/8/1778/s1.

## Abbreviations

vCCI	Viral CC Chemokine Inhibitor; also called 35K or vCCI/35K
vMIP-II	Viral macrophage inflammatory protein II
*E. coli*	*Escherichia coli*
NMR	Nuclear magnetic resonance
HSQC	Heteronuclear single quantum coherence
NaOP	Sodium phosphate
HHV-8	Human herpesvirus 8, Kaposi’s sarcoma-associated herpesvirus
MIP-1β	Macrophage inflammatory protein β, also known as CCL4
CCL4	CC chemokine ligand 4, also known as MIP-1β
IPTG	Isopropyl β-D-1-thiogalactopyranoside
DSS	4,4-dimethyl-4-silapentane-1-sulfonic acid
β-ME	β-mercaptoethanol
RMSD	Root mean squared deviation
RMSF	Root mean squared fluctuation (for specific residues)
MD	Molecular dynamics
