# Supplementary material for: Biophysical and Computational Studies of the vCCI:vMIP-II Complex
Source: Int J Mol Sci. 2017 Aug 16;18(8):1778. doi: 10.3390/ijms18081778 (PMC5578167; doi:10.3390/ijms18081778)
Supplement: Supplementary file 1 [file ijms-18-01778-s001.pdf]

Article

Biophysical and Computational Studies of the vCCI:vMIP-II complex

Anna F. Nguyen[1], Nai-Wei Kuo[1], Laura J. Showalter[1], Ricardo Ramos[1], Cynthia M. Dupureur[2], Michael E. Colvin[1], and Patricia J. LiWang[1*]

[1]Departments of Molecular Cell Biology and Chemistry, and the Health Sciences Research Institute, University of California Merced 5200 North Lake Rd, Merced, CA 953402

[2]Department of Chemistry & Biochemistry, University of Missouri-St. Louis, St. Louis, Missouri 63121

* To whom correspondence should be addressed pliwang@ucmerced.edu; (209) 228-4568

Supplementary Figures

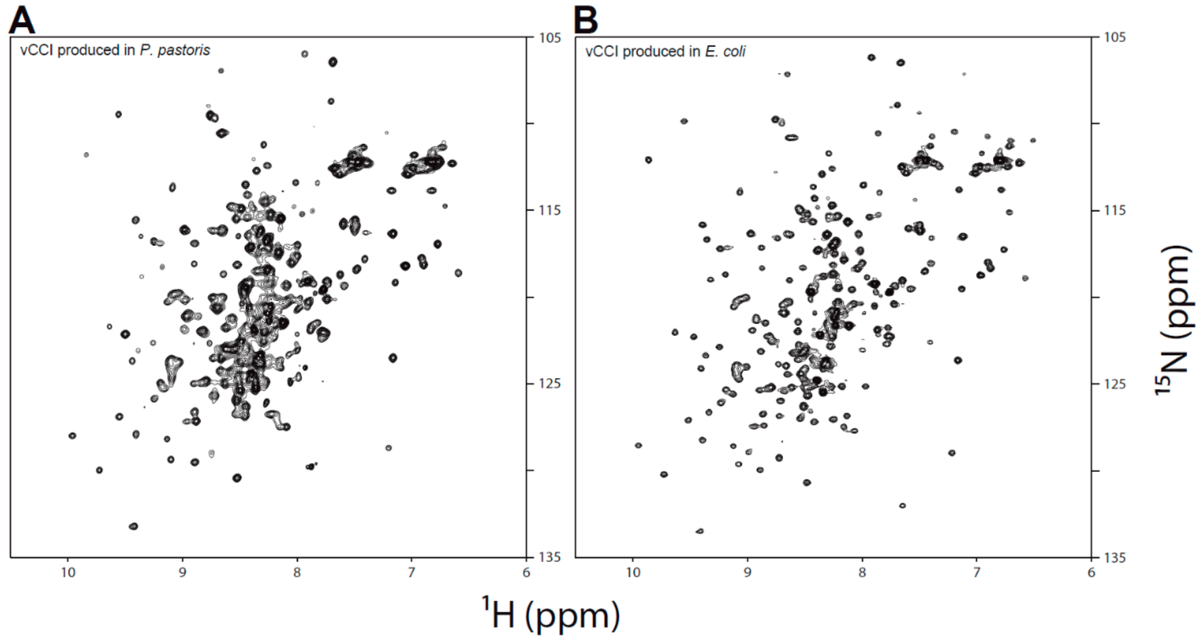

Supplementary Figure 1: Comparison of unbound vCCI produced in yeast and *E. coli.* (**A**) Uniformly side-chain deuterated $^{2}H/^{13}C/^{15}N$-labeled vCCI produced in *p. pastoris* yeast. Spectrum was measured in 100 mM NaCl, 20 mM NaOP pH 7.0, at 37°C, sample taken from [1]. (**B**) ^{1}H-^{15}N HSQC spectra of ^{15}N-labeled vCCI produced in *E. coli*, measured under the same conditions as in (A).

Supplementary Table 1: Results of isothermal titration calorimetry, titrating vMIP-II into a solution containing vCCI.

ΔH (kJ/mol)		ΔS (J/mol)		ΔG (kJ/mol)		n		K_D (M)	
Avg[1]	SD	Avg[1]	SD	Avg[1]	SD[2]	Avg[1]	SD	Avg[1,3]	SD[2]
-51.83	0.85	17.61	2.87	-57.08	NM	0.759	0.067	≤1.00E-10	NM

[1]The experiment was done in triplicate.

[2]NM: not meaningful. The values were the same, thus there was no apparent standard deviation.

[3]1.00E-10 is the low limit of K_D detection for the ITC.

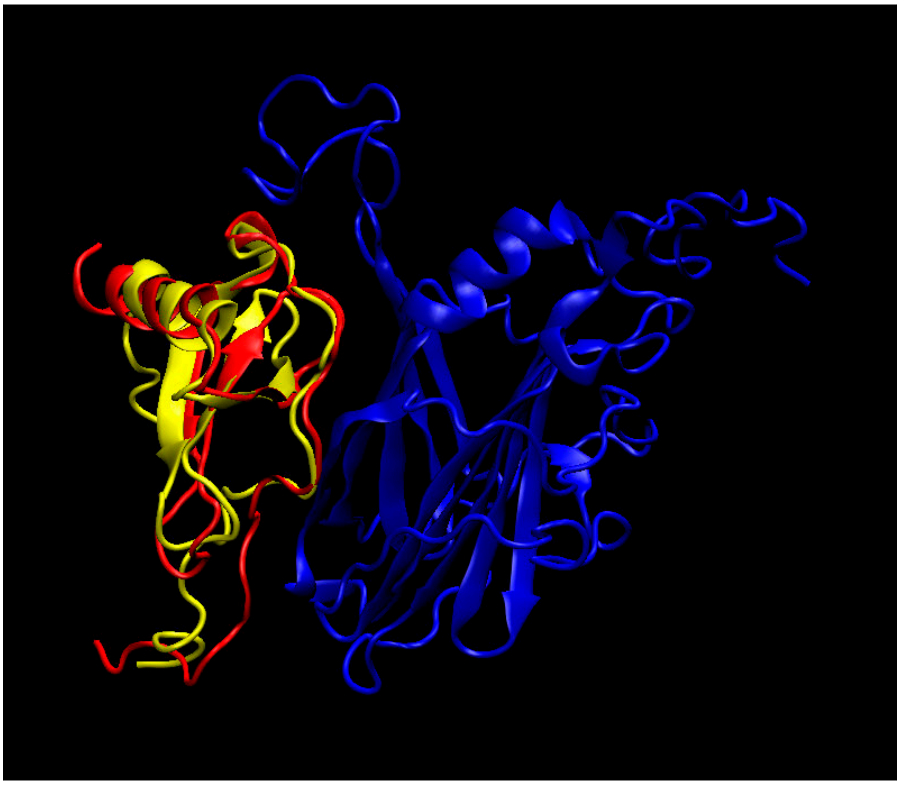

Supplementary Figure 2: vCCI:vMIP-II starting point for MD simulations. vCCI is shown as blue ribbon; vMIP-II is shown as yellow ribbon; MIP-1β is shown as red ribbon. The vCCI:vMIP-II trajectory was based on the vCCI:MIP-1β structure (PDB 2ffk; [2]) but the MIP-1B was replaced with the experimental structure of vMIP-II (PDB 1vmp), superimposed to minimize the difference in positions of the backbone atoms. This image was made in VMD[3].

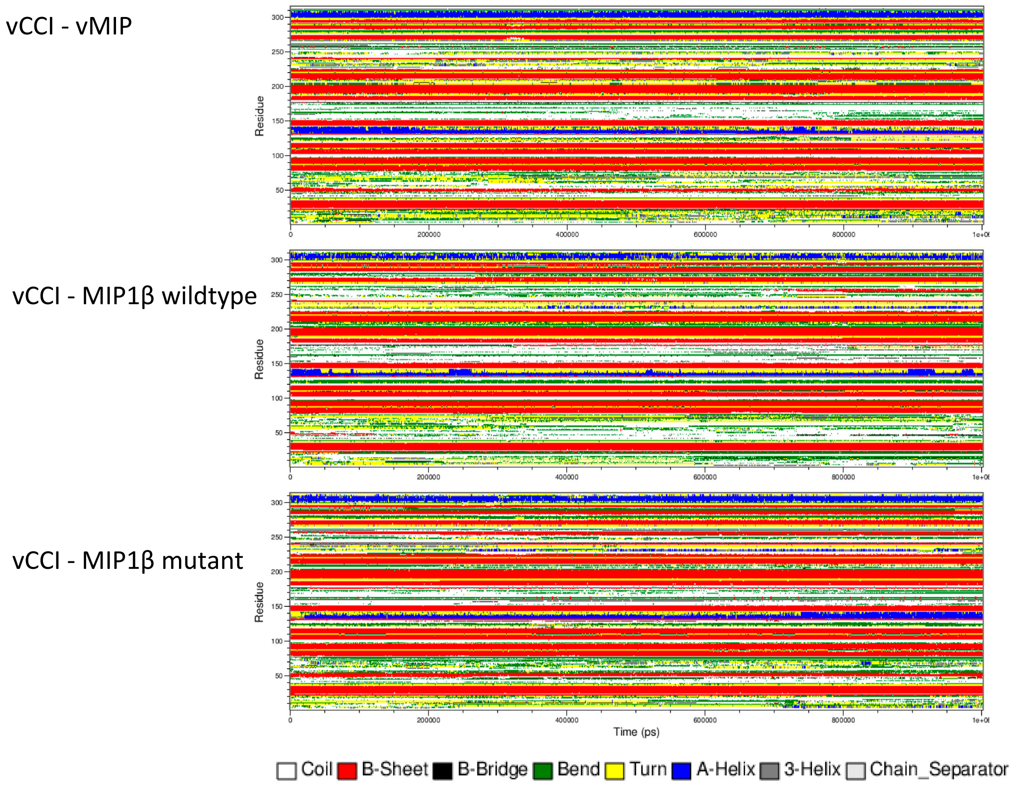

Supplementary Figure 3: Secondary structure of the complexes vCCI:vMIP-II, vCCI:MIP-1b, and vCCI:MIP-1β-K45A/R46A/L48A throughout the MD trajectory[4]. The y-axis shows the residue number in the complex, where residue 1 through 242 is vCCI and 243-313 (vMIP-II) or 243-311 (MIP-1β) is the chemokine.

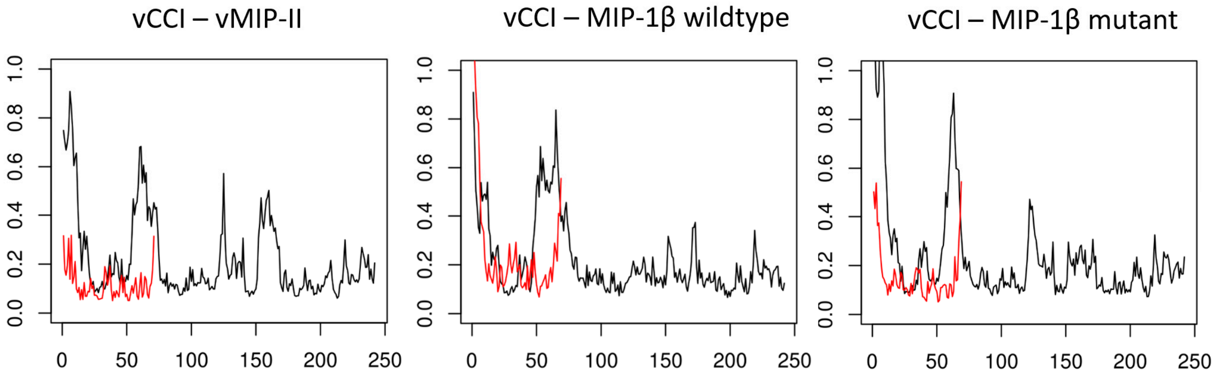

Supplementary Figure 4: Root mean square fluctuation values (in units of nm) plotted vs sequence location and calculated for the final 750 ns of the trajectories. The red line represents the chemokine, while the black line represents vCCI. Higher values show regions of increased protein flexibility, including both backbone and side chain motion.

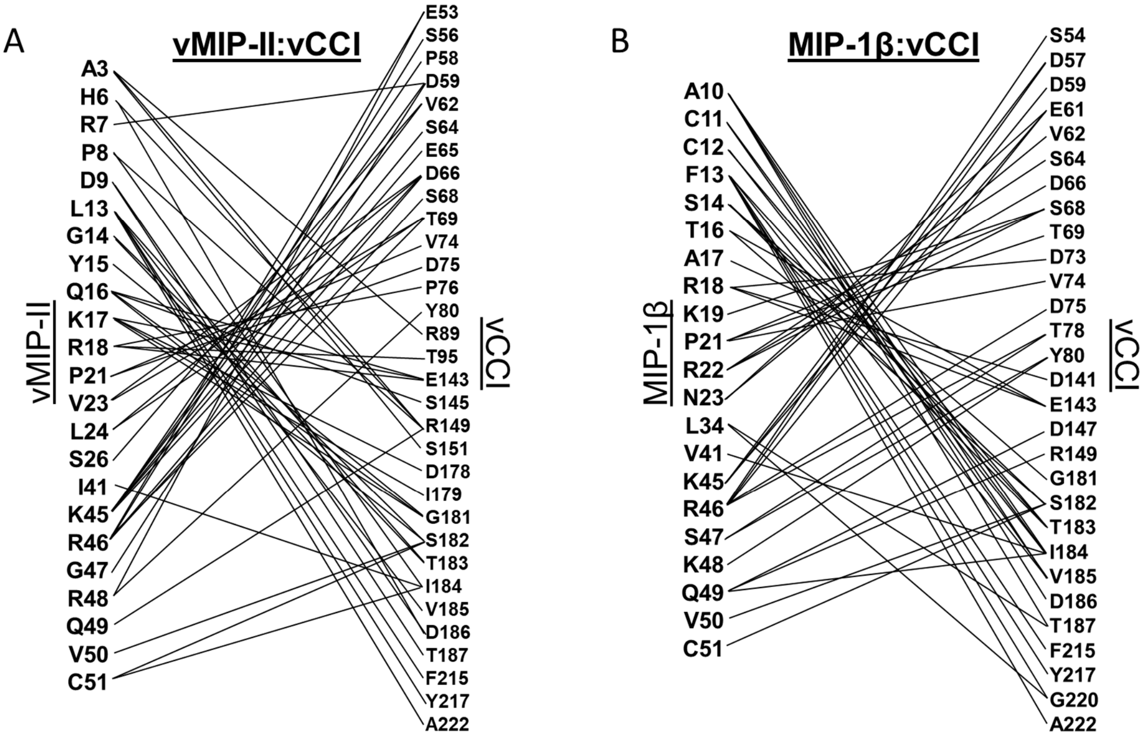

Supplementary Figure 5: (**A**) Interaction maps between residues of vCCI and vMIP-II and (**B**) interaction maps between residues of vCCI and MIP-1β. Interactions are shown for residues that are occluded during the simulation upon complex formation at least 50% of the time, and that are within 2.8 Å of the partner residue in at least 50% of the structures sampled every 20ns for the final 500ns of the trajectory.

References:

1. Zhang, L. Structural Study of the Interaction Between Poxvirus-Encoded CC Chemokine Inhibitor VCCI and Hhuman MIP-1β: A Dissertation. **2008**.
2. Zhang, L.; DeRider, M.; McCornack, M. A.; Jao, S. -c.; Isern, N.; Ness, T.; Moyer, R.; LiWang, P. J. Solution structure of the complex between poxvirus-encoded CC chemokine inhibitor vCCI and human MIP-1beta. *Proc. Natl. Acad. Sci.* **2006**, *103*, 13985–13990, doi:10.1073/pnas.0602142103.
3. Humphrey, W.; Dalke, A.; Schulten, K. VMD - Visual Molecular Dynamics. *J. Molec. Graphics* **1996**, 14, 33-38.
4. Kabsch, W.; Sander, C. Dictionary of protein secondary structure: Pattern recognition of hydrogen-bonded and geometrical features. *Biopolymers* **1983**, *22*, 2577–2637, doi:10.1002/bip.360221211.
